# Trans-population graph-based coverage optimization of allogeneic cellular therapy

**DOI:** 10.3389/fimmu.2023.1069749

**Published:** 2023-05-05

**Authors:** Sapir Israeli, Elizabeth F. Krakow, Martin Maiers, Corinne Summers, Yoram Louzoun

**Affiliations:** ^1^ Department of Mathematics, Bar-Ilan University, Ramat Gan, Israel; ^2^ Clinical Research Division, Fred Hutchinson Cancer Center, Seattle, WA, United States; ^3^ Department of Medical Oncology, University of Washington, Seattle, WA, United States; ^4^ Department of Bioinformatics, Center for Blood and Marrow Transplant Research, Minneapolis, MN, United States; ^5^ Department of Bioinformatics, National Marrow Donor Program/Be The Match, Minneapolis, MN, United States; ^6^ Pediatric Hematology/Oncology Department, Seattle Children’s Hospital, Seattle, WA, United States

**Keywords:** coverage, HLA, NK, T cell, model, therapies, frequencies, allogeneic

## Abstract

**Background:**

Pre-clinical development and in-human trials of ‘off-the-shelf’ immune effector cell therapy (IECT) are burgeoning. IECT offers many potential advantages over autologous products. The relevant HLA matching criteria vary from product to product and depend on the strategies employed to reduce the risk of GvHD or to improve allo-IEC persistence, as warranted by different clinical indications, disease kinetics, on-target/off-tumor effects, and therapeutic cell type (T cell subtype, NK, etc.).

**Objective:**

The optimal choice of candidate donors to maximize target patient population coverage and minimize cost and redundant effort in creating off-the-shelf IECT product banks is still an open problem. We propose here a solution to this problem, and test whether it would be more expensive to recruit additional donors or to prevent class I or class II HLA expression through gene editing.

**Study design:**

We developed an optimal coverage problem, combined with a graph-based algorithm to solve the donor selection problem under different, clinically plausible scenarios (having different HLA matching priorities). We then compared the efficiency of different optimization algorithms – a greedy solution, a linear programming (LP) solution, and integer linear programming (ILP) -- as well as random donor selection (average of 5 random trials) to show that an optimization can be performed at the entire population level.

**Results:**

The average additional population coverage per donor decrease with the number of donors, and varies with the scenario. The Greedy, LP and ILP algorithms consistently achieve the optimal coverage with far fewer donors than the random choice. In all cases, the number of randomly-selected donors required to achieve a desired coverage increases with increasing population. However, when optimal donors are selected, the number of donors required may counter-intuitively decrease with increasing population size. When comparing recruiting more donors vs gene editing, the latter was generally more expensive. When choosing donors and patients from different populations, the number of random donors required drastically increases, while the number of optimal donors does not change. Random donors fail to cover populations different from their original populations, while a small number of optimal donors from one population can cover a different population.

**Discussion:**

Graph-based coverage optimization algorithms can flexibly handle various HLA matching criteria and accommodate additional information such as KIR genotype, when such information becomes routinely available. These algorithms offer a more efficient way to develop off-the-shelf IECT product banks compared to random donor selection and offer some possibility of improved transparency and standardization in product design.

## 1 Introduction

Immune effector cell therapy (IECT) products are used for a variety of therapies for cancers and viral infection. “Off-the-shelf” refers to the ability to leverage healthy donors for on-demand or, more commonly, cryopreserved IECT products. A proliferation of published and ongoing trials attests to increasing interest in off-the-shelf allo-IECTs for anti-viral and anti-neoplastic indications. (See **Tables 1**, **S1** for a detailed list of proposed therapies.) Far more allo-IECTs are in preclinical development, as reviewed by Depil et al. (42) and Perez et al. (43).

**Table 1:** List of clinical trials of alloreactive immune effector cellular therapies. We list for each trial its reference, the type of disease treated, the cell type, the HLA constraint, and the target cell surface antigen or virus. Additional details are given in the **Supplementary Tables**.

Key Published Trials of Allogeneic Immune Effector Cell Therapy				
Reference	Disease Indication	Methodology		
		Cell type	HLA constraints	Target/Manufacturing N
Liu et al. (1)	B-cell lymphoma and CLL	Cord-blood-derived NK cells	Partial matching for the first 9 subjects, then enrolled with no regard to matching	CD19 targeting CAR, IL15, iCASP9
Benjamin et al. (2)	B ALL	T cells	Not stated	CD19 targeting CAR, TCR KO, CD52 KO
Mailankody et al. (3)	MM	T cells	Not stated	BCMA targeting CAR, TCR KO, CD52 KO
Lekakis et al. (4)	B-cell lymphoma	T cells	Not stated	CD19 targeting CAR, TCR KO, CD52 KO
Neelapu et al. (5)	B-cell lymphoma	T cells	Not stated	CD19 targeting CAR, TCR KO, CD52 KO
Quach et al. (6)	B-cell lymphoma	T cells	Best of HLA class I and II matching	CD30 targeting CAR, EBV-specific TCR
Bachanova et al. (7)	B-cell lymphoma and CLL	IPSC line-derived NK cells	Not stated	CD19 targeting CAR, CD16 Fc receptor, IL15/IL15 receptor fusion
Patel et al. (8)	B-cell lymphoma	IPSC line-derived NK cells	Not stated	CD19 targeting CAR, CD16 Fc receptor, IL15/IL15 receptor fusion
Jain et al. (9)	B ALL	T cells	Not stated	CD19 targeting CAR
Vasu et al. (10)	AML/MDS	NK cells	HLA and KIR genotyping (not further described) with demonstration of *in vitro* expansion	
Al-Homsi et al. (11)	MM	T cells	Not stated	BCMA targeting CAR, TCR KO
Ramos et al. (12)	B-cell lymphoma and ALL	NK T cells	Not stated	CD19 targeting CAR, IL-15, and shRNA targeting beta-2 microglobulin and CD74
Li et al. (13)	T cell ALL	T cells	Not stated	CD7 targeting CAR, CD7 KO, TCR KO
Holstein et al. (14)	MM	Placental CD34+ cell-derived NK cells	Not stated	
Cooley et al. (15)	AML/MDS	Placental CD34+ cell-derived NK cells	Not stated	
Kistler et al. (16)	Breast cancer	NK cells	Not stated	
Hu et al. (17)	B-cell ALL	T cells	Not stated	CD19/CD22 targeting CAR, TCR KO, CD52 KO
Patel et al. (8) and Hong et al. (18)	Solid tumors and lymphoma	iPSC-derived NK cells	Not stated	
Qasim et al. (19)	B-cell ALL	T cells	Not stated (“mismatched”)	CD19 targeting CAR, TRAC KO, CD52 KO
Tzannou et al. (20)	CMV post-HCT	T cells	≥ 2 of 8 shared HLA antigens	Peptide stimulation: IE1, pp65
Withers et al. (21)	CMV, ADV, or EBV post-HCT	T cells	≥ 1 of 6 shared HLA-antigens (-A, -B, -DRB1); highest number of HLA matches with antiviral activity through the shared HLA antigen(s). Secondary preference to products with highest virus-specific MHC-tetramer CD8+ cells or IFN γ response.	Peptide stimulation: pp65, AdV5 Hexon, BZLF1, LMP2, EBNA1
Leen et al. (22)	CMV, ADV, or EBV after HCT	T cells	Specificity for the target virus through a shared HLA allele. Secondary preference to maximize HLA matches.	Transduction with Ad5f35pp65 multivirus-specific vector
Haque et al. (23), Haque et al. (24) and Haque et al. (25)	PTLD after HCT or SOT	T cells	Maximize HLA match out of 6 (HLA-A, B, DR), with ≥ 1 HLA-A and ≥ 1 HLA-B match. Secondary preference to CTLs with the highest cytotoxicity in chromium release assays against patient LGLs and low killing of patient PHA blasts, mismatched LCLs, and K562.	Sensitization by EBV-BLCLs
Neuenhanh et al. (26)	CMV after HCT	T cells	≥ 1 shared HLA class I allele that can restrict the CMV-specific target	Direct isolation: MHC-Streptamer purification of CMV epitope-specific T cells from unstimulated donor leukapheresis
Tzannou et al. (27)	CMV, ADV, EBV, BK, or HHV-6 after HCT	T cells	Specificity for target virus through shared HLA alleles. Secondary preference to overall HLA match. Used epitope mapping, cytokine profiling and cytotoxicity to confirm antiviral activity through ≥ 1 shared HLA allele prior to selecting a VST line.	Peptide stimulation: IE1, pp65, Hexon, Penton, EBNA1, LPM2, BZLF1, VP1, large T, U11, U14, U90
Doubrovina et al. (28)	PTLD after HCT	T cells	Selection based on: (a) Cytotoxicity assessed against autologous donor- and patient-derived EBV+ BLCL and EBV− PHA blasts, and (b) against a panel of allogeneic EBV-BLCL, each matching one of the HLA alleles expressed by the T cells. cf. Comments.	Sensitization by EBV-BLCLs
Feuchtinger et al. (29)	CMV after UCB	T cells	Not stated	Peptide stimulation: pp65, enriched for IFN γ secretion
Barker et al. (30)	PTLD after UCB	T cells	Cytotoxicity in chromium release assay against a panel of EBV+ and EBV− targets expressing one set of HLA A, B, C, DR, and DQ alleles shared by the CTL donor. CTLs with the closest HLA match to the UCB unit (and hence the lymphoma) restricted by one or more of the CTL donor’s HLA alleles, and HLA match > 2/10 to the patient, was selected.	Sensitization by EBV-BLCLs
Prockop et al. (31)	PTLD after HCT or SOT	T cells	HLA type, immune phenotype, lack of alloreactivity, EBV-specific cytotoxicity, and HLA restriction as per Doubrovina et al. (28).	Sensitization by EBV-BLCLs
Papadopoulou et al. (32)	CMV, ADV, EBV, BK, or HHV-6 after HCT	T cells	Specificity for target virus if reactivation/infection (*vs*. prophylaxis)	Peptide stimulation: IE1, pp65, Hexon, Penton, EBNA1, LPM2, BZLF1, VP1
Moftuoglu et al. (33)	PML	T cells	Most closely matched (minimum requirement: ≥ 1 HLA class I and ≥ 1 HLA class II allele match)	Peptide stimulation: VP1, VP2, VP3, ST, LT
Sun et al. (34)	EBV+ Hodgkin lymphoma	T cells	Minimum ≥ 3/6 HLA match	Sensitization by EBV-BLCLs
Gallot et al. (35)	PTLD after HCT or SOT, EBV+ lymphoma after autologous HCT	T cells	≥ 1 match for HLA class 1 and EBV specificity through a shared HLA allele (priority); negative cytotoxicity test against the patient’s PHA blasts; EBV-CTLs cytotoxicity score > 15% against the autologous EBV-LCLs and >2× that observed against patient PHA blasts	Sensitization by EBV-BLCLs
Naik et al. (36)	Primary immunodeficiency: EBV or CMV prior to or after HCT; PTLD	T cells	Various strategies. Donors were 3/10 to 9/10 matched to patients.	Various
Vickers et al. (37) and Kazi et al. (38)	Primary immunodeficiency with EBV pre-HCT; PTLD or EBV after HCT or SOT	T cells	Maximize HLA class I and II matches, then minimize the number of mismatches.	Sensitization by EBV-BLCLs
Chiou et al. (39)	PTLD after SOT	T cells	Maximize HLA match (pre-2005—out of 6 loci; post-2005—out of 10 loci)	Sensitization by EBV-BLCLs
Fabrizio et al. (40)	CMV after HCT	T cells	Not explicitly stated	Peptide stimulation: 15-mer overlapping peptides spanning pp65
Jiang et al. (41)	CMV, EBV after HCT	T cells	Maximize the number of HLA matches with antiviral activity through shared HLA antigen(s), out of 6. Secondary preference to the product with the highest proportion of virus-specific responses through shared allele(s).	Peptide stimulation: pp65 and EBV consensus peptides. Then, CD137+ cells were selected and sensitized in culture by peptide-pulsed CD137− cells.

The potential advantages of allogeneic over autologous IECT approaches include (a) immediate availability of cryopreserved product; (b) avoiding inadequate collection of starting material from patient leukapheresis due to lymphopenia or autologous T or NK cell dysfunction (due to the immunosuppressive effects of cancer or the extent of prior chemotherapeutic and immunomodulatory treatments); (c) avoiding treatment delays introduced by complex logistics and manufacturing failures; (d) possible improvements to standardization and dose–response prediction; (e) time for additional cell modifications that could increase efficacy, safety, or persistence; (f) ease of repeat dosing; and (g) economies of scale that can reduce the cost burden on healthcare systems and may increase accessibility of IECT worldwide.

On the other hand, allo-IECT faces several challenges, including the risk of graft-*vs*-host disease (GvHD) and the rapid elimination of the cell product by recipient NK or T cells (Depil et al. (42). GvHD occurs when the donor-derived T cells attack the recipient’s healthy tissue. This donor cell reaction is associated with HLA molecules on the recipient tissue that are not expressed in the donor. Conversely, the host can reject the target cells, when foreign HLA molecules on the donor-derived cells trigger the recipient’s T cells to react against the donor-derived cells. Alternatively, recipient NK cells can react against donor cells that are missing an HLA molecule native to the recipient. These are reasons why IECTs with “HLA independent” mechanisms of anti-viral or anti-cancer efficacy may still benefit from consideration of HLA compatibility. Strategies for overcoming these challenges are described in the **Supplementary Tables**. For example, disrupting the TRAC locus to prevent TCR expression can eliminate the risk of GvHD in the context of donor T-cell therapies. Knocking out the beta2 microglobulin gene to prevent expression of class 1 HLA on donor T or NK cells may “hide” them from recipient T cells to increase persistence, but additional gene editing would be necessary to reduce the likelihood of lysis by recipient NK cells noticing a “missing self” ligand. A chimeric 4-1BB-specific alloimmune defense, proposed by Mo et al., enables CAR-T cells to evade alloreactive recipient T and NK cells, yet spares recipient resting T and NK cells. This avoided immunocompromise and promoted persistence and anti-tumor efficacy (44).

The optimization strategies for choosing a set of candidate donors consistent with the challenges described in **Table S2** depend on the clinical context and the extent of genetic engineering deemed feasible. Foremost is the indication for therapy. For example, IECT may safely be rejected after clearance of an infection with no latent form but may need to persist for recurring infections. Similarly, if a tumor is rendered operable by neoadjuvant IECT debulking, long-term IEC persistence may be superfluous after successful tumor resection. However, IEC persistence may be essential in situations where sub-clinical malignancy may lead to relapse. We must also consider the anticipated adverse effects of IEC persistence due to on-target/off-tumor effects, such as B-cell aplasia for CD19+ ALL or myeloid aplasia for CD123-directed CAR-T cell therapy.

To support emerging efforts at product standardization and to maximize population coverage while minimizing costs associated with collecting redundant donors, we propose a solution to the maximal coverage problem for different scenarios and compare the optimal coverage with the one obtained from random donors. These algorithms could accommodate information beyond HLA typing, such as KIR genotyping or polymorphisms in other immune response genes.

## 2 Methods

### 2.1 Genotype data

The datasets obtained from the Ezer-Mizion Bone Marrow Donor Registry include 1,040,503 donors. The population HLA haplotype frequencies were estimated using a multi-race expectation-maximization algorithm (45). The HLA of each donor was imputed using GRIMM (46) and the most probable five locus (A, B, C, DQB1, and DRB1) genotypes were chosen.

### 2.2 Problem goal

Given two sets of genotypes, R1 and R2 , of donors and patients, respectively, each with 2 mismatch rates, a and b, we say that genotype j ( $d_{j}$ ) of donors matches genotype i ( $g_{i}$ ) of patients if it obeys some matching condition—for example, at most a mismatches in class 1 and b mismatches in class 2. The goal is to find the minimal set of donor genotypes that optimizes the patients’ coverage.

The method includes two stages.

1. Find for each donor genotype $d_{j}$ in the donor population ( R1 ) which patient genotypes it can match (further denoted as $S_{j}$ ).
2. Assuming a weight $p_{i}$ for each genotype in a patient population ( R2 ), which represents the number of patients with the same genotype, the problem can be stated in two similar ways: (A) Given a maximal size of the set of donors r1⊂R1,|r1|=N , find the subset of donor genotypes that maximizes $\sum_{i}p_{i}|\left(g_{i}\in S_{j},d_{j}\in r1\right)$ , or alternatively, (B) for a required coverage $P=\sum_{i}p_{i}|\left(g_{i}\in S_{j},d_{j}\in r1\right)$ , find the minimal subset that produces a coverage of P.

### 2.3 Graph model—Stage 1

For each genotype *d_j_* in the donor population ( R1,j=1,2…,N1 ), create a node of the full unphased genotype (denoted *UMUG*—Unphased Multilocus Unambiguous Genotype) and then create edges from the genotype node to the appropriate class 1 and class 2 nodes, *C*1*_j_* and *C*2*_j_*. A *Ck_j_* node is composed of a pair of class *k* genotypes (e.g., $A1+A2\;\hat{\;}B5+B8\;\hat{\;}C12+C3$ for the appropriate set of genes A,B,C ). Here, we use a two-field representation of the alleles (e.g., A*02:01 ).

**Figure 1:**
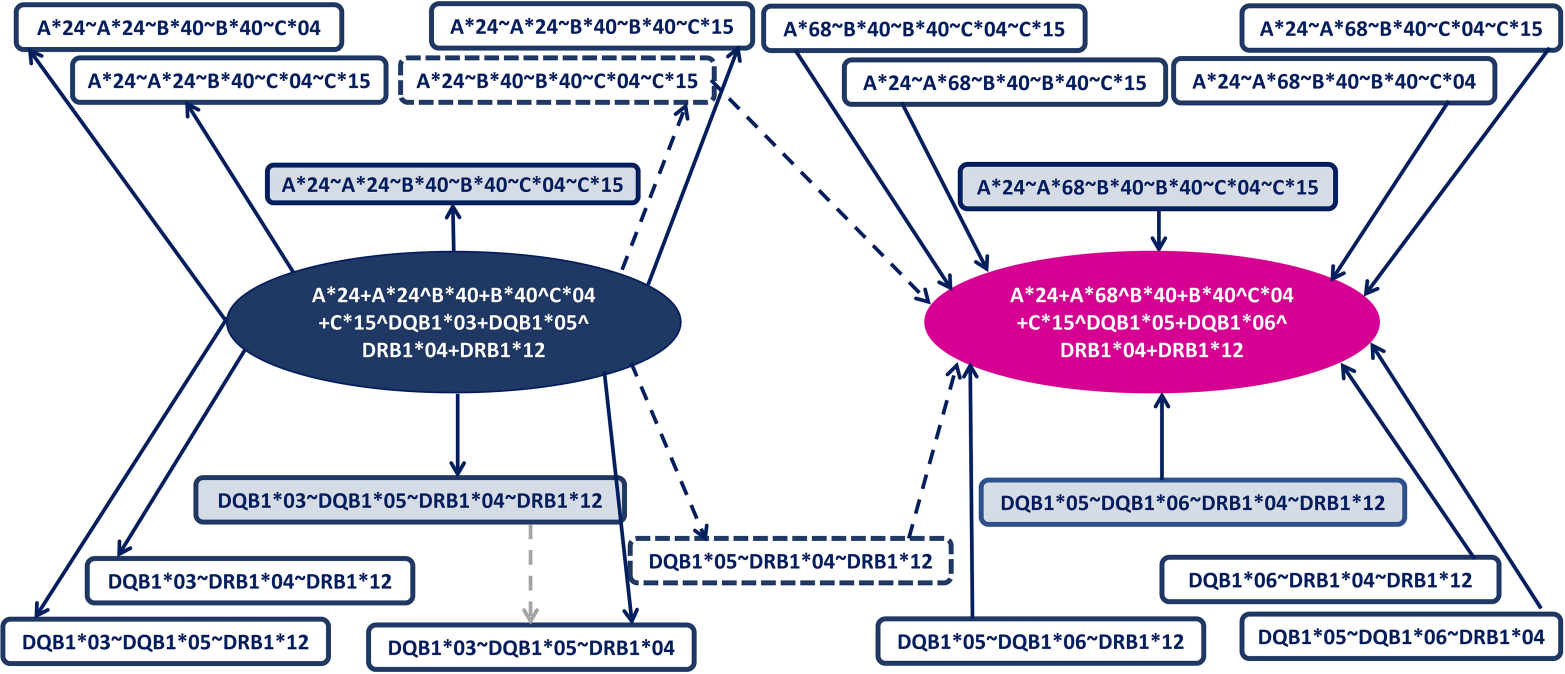
Example of graph creation. Here, we allow one mismatch in both class 1 and class 2. For the donor genotype (dark blue) and the patient genotype (pink), a sub-node of class 1 and class 2 (gray-blue nodes) was created, and then the sub-node of class 1 minus 1 and class 2 minus 1 (white nodes) was created. Each sub-node was connected to the corresponding genotype nodes. The dashed gray edge shows that the white node is a sub-node of the gray-blue node, but those edges do not exist in the graph. If there exists a path between two nodes that passes through class 1 sub-nodes and through class 2 sub-nodes, then those nodes cover each other. Here exist two paths (the dashed path).

First, merge all patient genotypes and save the number of occurrences. For $Ck_{j}$ , with z alleles, create the combination of all z−1 alleles $Ck_{l}$ , and create edges from the full genotype (e.g., $A1+-\;\hat{\;}B5+B8\;\hat{\;}C12+C3$ in the example above). Repeat the iterative process, starting from $Ck_{l}$ , until z−a alleles for class 1 and z−b alleles for class 2. For the patient genotypes, we create the same connection but with opposite edge direction (**Figure 1**). The weight of patient genotype vertex is the number of genotype occurrences.

Given two sets of donor and patient genotypes, R1 and R2 of size N1 and N2, respectively, for each genotype $d_{j}$ from R1 , define $S_{j}$ to be all the genotypes from R2 (reachable from $d_{j}$ through the graph); the problem can be stated as the maximal coverage of R1 by the union of the $S_{j}$ .

### 2.4 Optimal coverage

**Linear programming**: This problem can be formulated as an LP problem (47): $x_{j}$ is a binary flag that represents whether a donor with genotype $d_{j}$ was chosen in the cover ( r1 ). $y_{i}$ represents whether patient genotype $g_{i}$ is covered by r1 . We define a loss function


(1) $$Loss=\sum^{}x_{j},$$

and minimize it subject to:


(2) $$\sum_{i\in N2}d_{i}y_{i}=P$$

(3) $$\sum_{g_{i}\in S_{j}}x_{j}\geq y_{i}$$

(4) $$y_{i}\in \left[0,1\right]$$

(5) $$x_{j}\in \left[0,1\right]$$

**Integer linear programming**: For ILP, replace the last two with:


(6) $$y_{i}\in \left\{0,1\right\}$$

if $y_{i}=1,$ then $g_{i}$ is covered.


(7) $$x_{j}\in \left\{0,1\right\}$$

if $x_{j}=1,$ then $S_{j}$ is selected for the cover.

**Greedy algorithm**: The greedy algorithm (48) at each iteration chooses set v[i] that contains the maximum weight of uncovered elements until the wanted percentage is covered.

## 3 Results

### 3.1 Optimal coverage

To estimate the optimal population coverage for population R2 that can be obtained using a set of donor cells from population R1 (that can be the same or different populations), one can compute a coverage problem. Each person i in population R2 is characterized by an HLA genotype $g_{i}$ and a probability $p_{i}$ that represents the number of patients who may require treatment (or a preference) whose HLA genotype is $g_{i}$ . The goal is to find a minimal subset of donors from population $\left\{d_{j}\in r_{1}\subset R1\right\}$ , such that the fraction of the population in R2 that can receive a treatment from them is maximal. We can define for each donor j the set of all patients who can receive a treatment from this donor $S_{j}$ .

Formally, we try to find the subset r1 that maximizes:


(8) $$max(\sum_{i}p_{i}|g_{i}\in S_{j},d_{j}\in r1)$$

Note that the same person can receive treatments from different donors, such that different $S_{j}$ may overlap. The definition of $S_{j}$ is determined by the treatment proposed, and may differ drastically between treatments. We have tested three protocols, with large differences between the resulting optimal number of donors depending on the treatment.

1. The donor is KIR-Bw4 mismatched to the patient and requires a full match in class 2, while no match is required in class 1.
2. The donor and the patient have a maximal match at the HLA-A and HLA-B loci. The patient and the donor must both have A*02:01 and the donor must not be homozygote in any HLA allele shared with the patient.
3. All A, B, C, DRB1, and DQB1 alleles that appear in the donor should also be in the patient. The opposite does not have to happen. For example, the donor may be homozygous at a locus where the patient is heterozygote. In the case of mismatch, a knockout for one of the donor alleles can be performed, but at a high cost (which is equivalent to using more donors with no knockout). In this case, we aim at optimizing the cost and not the total number of donors.

To compute the optimal donor set for large populations, one must first compute efficiently the coverage of each donor ( $S_{j}$ ) and then solve the optimization problem. We propose novel solutions for each stage. The $S_{j}$ computation is performed through an extension of the GRIMM graph matching Maiers et al. (46). The second is solved through a linear programming problem.

### 3.2 Optimal coverage computation

We developed a graph-based algorithm to solve the following problem: Given a set of patients, each with a genotype $g_{i}$ , a donor with a genotype $d_{j}$ , and 2 mismatch rates, a and b, we look for the set of patients who have at most a mismatches in class 1 and b mismatches in class 2. The genotypes covered can be obtained through a traversal in that graph (see *Section 3.3* and **Figure 1**).

Given the coverage obtained by the graph, one can solve the optimization problem in Eq. 1 using four possible methods.

- A greedy solution, where, at each stage, the donor *j* provides the largest coverage of the remaining population.
- A linear programming (LP) solution, where a GPLK algorithm (49) is used. The LP provides partial fraction for each donor. As such, it cannot be used in practice (since one cannot take half a donor). This solution is an upper bound for the optimal solution. We further show that the greedy and ILP results are similar to the LP.
- Integer linear programming (ILP). We used the CBC algorithm (50). This is the best theoretical solution.
- The random choice of donor. We computed the average coverage of five random choices of *N* donors.

### 3.3 Scenarios

#### 3.3.1 Scenario 1: NK cell therapy

An off-the-shelf NK cell therapy is being developed to treat myeloid malignancies, as in Lamb et al. (51). It is hypothesized that if the patient is missing a ligand (HLA) for which the donor possesses the cognate KIR, some donor NK cells may be uninhibited upon contact with malignant cells, improving the donor-*vs*-leukemia effect. It is further hypothesized that maximizing the class 2 HLA matching will improve donor cell persistence. The limitations on the donors in this scenario are as follows:

1. Donor is KIR-ligand mismatched to the patient.
2. Full match in class 2.
3. Six mismatches can be allowed in class 1.

For the KIR mismatched limitation, we used Bw4 expressed on HLA A or B with 0–4 appearances. Two genotypes match in KIR if both have the same epitopes (regardless of the number of occurrences of each one). For class 2, we demanded no mismatch.

**Figure 2:**
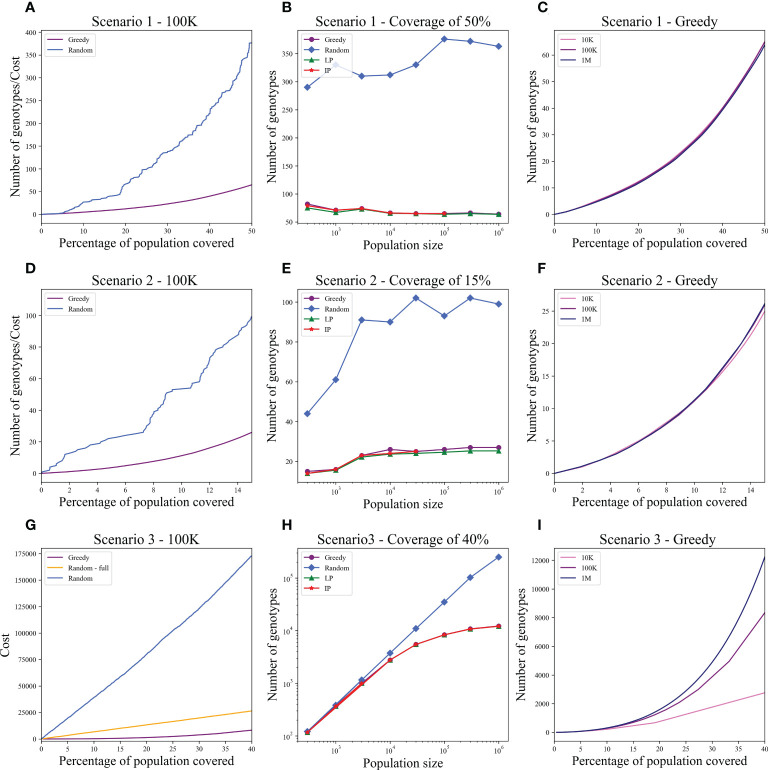
Coverage in different models. For each scenario described in the text, we checked how many donors are needed to cover the total population. Each row is a different scenario. **(A, D, G)** The cost to cover the given percentage from the population of size 100K (*x*-axis) using two algorithms: greedy and random choice. In G, options for random choice included the full genotype or knockout genotypes in each iteration (**“**Random**”**) or only full genotypes (**“**Random - full**”**). **(B, E, H)** How many genotypes are needed to cover 50% **(B)**, 15% **(E)**, and 40% **(H)** of differently sized populations represented on the ***x***-axis on a log scale. The *y*-axis is on a log scale in **(H). (C, F, I)** The number of genotypes needed (*y*-axis) to cover × percentage of the population (*x*-axis) in three different population sizes: 10K, 100K, and 1M.

**Figure 3:**
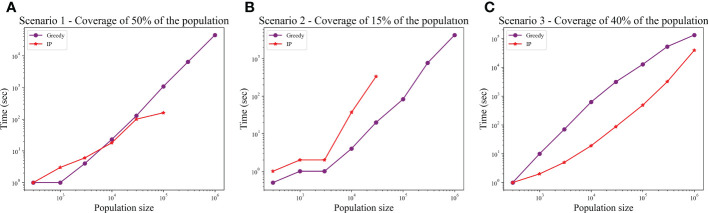
Comparison between runtime of IP and greedy algorithms. The graphs show the effect of the population size on the runtime, in each of the scenarios: **(A)** Scenario 1, **(B)** Scenario 2, **(C)** Scenario 3. In scenarios 1 and 2, the IP could not converge when populations were too large (the missing dots).

We computed the optimal coverage using a random choice, and compared it to the different optimizations (greedy algorithm, LP, and ILP) on a population of 100,000 patients and the same 100,000 donors, and required a coverage of at least 50% of the population. The greedy and ILP algorithm found similar minimal sets (**Table 2**), which are six times smaller than the random. We also compared how many genotypes are needed to cover different fractions of the population by the greedy and random choice (**Figure 2A**). We further compared the number of donors required to cover the population in the four algorithms for different populations sizes: 300, 1K, 3K, 10K, 30K, 100K, 300K, and 1M (**Figure 2B**). In large populations, the random solution requires more donors, whereas in the other algorithms, the number of donors required actually decreases with the patient population size. In a larger population, there is a greater chance of finding rare donors that match multiple patients, and thus fewer donors are actually needed. The patient population may be more heterogeneous, but we aimed to cover 50%, the algorithm. Thus, missing rare patients has a smaller effect than finding better donors. On the other hand, the number of donors required to cover an additional percent of the patient populations increases as more coverage is required. While 10 donors can cover 10% of the population, 40 donors are required to cover 40% (**Figure 2C**). For small populations, the runtimes of the greedy algorithms and ILP are similar. For large populations, ILP resolves faster, but for populations above 30,000, the ILP algorithm fails to converge following inherent limitations of the ILP algorithm (**Figure 3A**).

**Table 2:** Scenario 1. Comparison of four algorithms: random, greedy, LP, and ILP, to cover 50% from a population of 100K patients by 100K donors, where the patient population and the donor population are identical.

	Percentage of population covered	The number of genotypes needed	Runtime (sec)
Random	50.150.21	306.617.33	6
Greedy	50.03	65	1079
LP	50	63.67	202
ILP	50	65	154

The comparison includes how many genotypes are needed to cover 50% of the patient population, and the runtime of each algorithm.

#### 3.3.2 Scenario 2: Neoantigen-specific TCR T-cell therapy

A clinical bridge-to-transplant trial is open for patients with relapsed acute leukemias. Following chemotherapy, patients will receive off-the-shelf transduced TCR T-cell products specific for immunogenic leukemia-associated epitopes presented on HLA-A*02:01, such as *p*53^R175H^ (52) and W_T37−45_ (53). The limitations in this scenario are as follows:

1. The donor and the patient must both have HLA-A*02:01.
2. To minimize the risk of intractable GvHD, the TCR T-cell donor must not be homozygous at any HLA allele shared with the patient.
3. To minimize the risk of “too prompt” a rejection of the TCR T cells by patient NK cells, the donor and the patient should be matched as much as possible at HLA-A and HLA-B.

Only genotypes with HLA-A*02:01 were included in the graph. If $d_{j}$ is homozygote in any HLA allele, then we removed from $S_{j}$ all $g_{i}$ with those alleles. The graph was changed to contain sub-nodes of HLA-A and HLA-B instead of nodes of all class 1; sub-nodes of class 2 were removed.

For limitation 3, we implemented the greedy algorithm to find at least three matches with a priority to four, defined as a constant called $Prior_{4}$ . Assume patient genotypes of $g_{i}$ ; in each iteration, we want to choose the donor genotype $d_{j}$ that maximize:


(9) $$Loss=\left(\sum^{}p_{i}*Prior\left(i\right)|g_{i}\in S_{j}\right).$$

If the number of matches between $g_{i}$ and $d_{j}$ in HLA-A and HLA-B is 4, then $Prior\left(i\right)=Prior_{4}$ , else Prior(i)=1 .

Since one of the requirements of this scenario is to maximize the matches at HLA-A and HLA-B between the donors and patients, we tested how many donors are required for a full match in A and B, or for a match of at least three out of four. We used again the greedy, random, LP, and ILP algorithms. Beyond that, we implemented the greedy algorithm to find at least three matches with different priorities to four, as a function of $Prior_{4}$ .

We tested the four algorithms on a population of 100,000 patients and the same 100,000 donors; 24.956% of the population had at least one copy of A*02:01. We thus looked for a more limited coverage of at least 15% of the total population. For a match of three, the ILP failed to find a solution. The performance of the greedy with the priority to four provided a better solution compared to the regular greedy, the addition of three genotypes to cover a greater number of four matches. For a required full match in A and B, the greedy performance is equal to the ILP and LP, but much more genotypes are needed (more than 50 time more) compared with the model with only three out of four matches required in A and B or with the softer model where a preference is given to four matches (**Table 3**).

**Table 3:** Scenario 2. Comparison of four algorithms: random, greedy, LP, and ILP, to cover 15% from a population of 100K patients by 100K donors, where the patient population and the donor population are identical.

	Match	Prior_4_	Population covered	4 matches	3 matches	Genotypes needed	Runtime
Random	3M	–	15.08 ± 0.08	387.4 ± 109.4	14,695.9 ± 139.4, 96.1 ± 5.7		1.5
Greedy	3M	–	15.035%	502	14,533	26	83
Greedy	3M	2	15.007%	522	14,485	26	109
Greedy	3M	10	15.1%	1124	13,983	29	98
Greedy	3M	100	15.014%	1191	13,823	29	109
LP	3M	–	15%	2,433.34	12,566.66	24.61	34
Random	Full	–	15 ± 0.002	15,000 ± 2.2	–	5,752.2 ± 104.73	2
Greedy	Full	–	15%	15,000	–	1,565	310
LP	Full	–	15%	15,000	–	1,565	20
ILP	Full	–	15%	15,000	–	1,565	23

The comparison includes how many genotypes are needed to cover 15% of the patient population, and the runtime of each algorithm. Match— number of at least matches at HLA-A and HLA-B. $Prior_{4}$ —the priority size for a full match at A and B. Population covered—Percentage of population covered. 4 matches—number of genotypes in the cover, with 4 matching in HLA-A and HLA-B (the same for three matches). Genotypes needed—the number of genotypes needed for this cover.

We also compared how many genotypes are needed to cover different fractions of the patient population by the greedy and random choices (**Figure 2D**). In addition, we compared all the algorithms for different populations sizes: 300, 1K, 3K, 10K, 30K, 100K, 300K, and 1M (**Figure 2E**). The greedy performances are close to ILP and LP. Except for the random model, in all models, the number of required donors stabilizes between 1,000 and 10,000 patients (at less than 30 donors). The number of required donors is not affected by the population size for all coverage fractions tested (**Figure 2F**). For the one mismatch case, the runtime of the greedy algorithm is lower than the ILP, since we require a low coverage of the patient population, and it converges using less iterations (**Figure 3B**).

#### 3.3.3 Scenario 3: Polyclonal T-cell infusion

A clinical trial of alpha/beta depleted T-cell therapy for various malignancies (not post-HCT) is planned, as in NCT05001451 and others reviewed in Saura-Esteller et al. (54), and the risk of clinically significant GvHD with this product is deemed to be low. However, the researchers seek to maximize HLA matching as they hypothesize that this will increase donor T-cell persistence and the ability to respond to the cross-presentation of tumor-associated antigens, and improve efficacy. They are able to knock out single HLA alleles using gene editing, but it is expensive. They seek to identify the most cost-efficient way to build the cell product bank: Recruit more donors or remove mismatched HLA loci? The limitations in this model are that all alleles that appear in the donor should also be in the patient, with two options:

1. Full 10/10 HLA match (A, B, C, DRB1, and DQB1).
2. Knockout for one of the donor alleles, and match between the nine other alleles between the donor and the patient. A knockout solution costs like $Cost_{KO}$ regular donors ( $Cost_{KO}$ is a constant parameter). Formally, we minimize
(10) $$Loss=\left(\sum^{}x_{j}*COST_{j}\right)$$
  If $x_{j}$ represents a full genotype, then $COST_{j}=1$ , else *COST*_*j*_ = *Cost*_*KO*_ + 1.

We want to minimize the total cost for a given coverage of the patient population.

In the graph, we added all nine allele combinations of each donor genotype and created nodes similar to the full genotype nodes, extended to the class 1 and 2 nodes similarly. In this graph, the set R1 is larger than the number of donors (since we typically added 10 more nodes per donor). We thus improved the performance by connecting each node in R1 directly to matched nodes from R2 . In this scenario, the ILP is faster than the greedy and it always converges (**Figure 3C**).

In the greedy solution, at each iteration, we find the knockout genotype that covers the maximum number of patients ( $S_{K}$ ). Then, we find how many full donors ( $N_{G}$ ) are needed to cover at least such a number of patients. The total number of patients covered by the $N_{G}$ donors is $S_{F}>=S_{k}$ . If the average cost If the average cost of a patient coverage by a knockout ( $\frac{S_{K}}{Cost_{KO}+1}$ ) is smaller than the cost of a patient with a regular donor ( $\frac{S_{F}}{N_{G}}$ ), we choose the knockout solution for this iteration, else we choose the full genotype solution.

Using the greedy algorithm, we tested how many full genotypes and knockout genotypes are needed to cover 25% and 40% from populations in size 50K and 100K when the knockout price is 5- and 10-fold the full genotype. When the price is higher by 10-fold, the knockout does not pay off (**Table 4**). We compare the greedy and the random choice, when the random can choose full genotype or knockout genotype in each iteration, and when the random can choose only full genotypes. As mentioned, the greedy chose only full genotypes. Full genotypes are preferable when the cost is equal to 10-fold (**Figure 2G**). For a coverage of 40% of the population, the greedy always chooses full genotypes while the ILP chooses a few knockout genotypes that grew with the population size (**Table 5**), but in comparing the four algorithms, it can be seen that the greedy LP and ILP have a similar performance while the random choice is very expensive (**Table 6**). Also, as the population size increases, so does the number of genotypes needed (**Figure 2H**). The last also occurs for coverage of less than 40%. For cost equal to 10-fold, we check the number of genotypes needed to cover each percentage from the population for populations of size 10K, 100K, and 1M. It can be seen that for a small percentage of the population, one genotype can cover a greater number of genotypes and therefore the ratio between the number of genotypes that cover and genotypes successfully covered increases as the percentage of the population increases (**Figure 2I**). All the above-mentioned genotypes are for a donor population identical to the patient population, where, in general, the knockout yields less payoff, but when the populations are different, from a certain percentage of population coverage, full genotypes cannot be matched and the knockout solution must be used (**Table 5**).

**Table 4:** Scenario 3. Greedy algorithm outcomes of different combinations of population size, price, and percentage of the population to cover.

Population size	Price	Population covered	Total genotypes number	Knockout genotypes number
50K	5	25%	2,106	1
50K	5	40%	6,315	29
50K	10	25%	2,116	0
50K	10	40%	6,472	0
100K	10	25%	2,406	0
100K	10	40%	8,339	0

Price—By how much the price of a knockout is greater than a full genotype. Population covered—Percentage of population covered. Total genotypes number—the number of knockouts and full genotypes that need to cover. Knockout genotypes number—the number of only knockout genotypes that participate in the cover.

**Table 5:** Scenario 3. ILP outcomes of different population sizes to cover 40% of the patient population, for identical donor and patient populations, compared to different donor and patient populations. where the cost of full is 1 and knockout is equal 10 + 1.

Size	Identical populations			Different populations		
	Total Cost	Knockout	Full	Total Cost	Knockout	Full
300	117	0	117	1,240	112	8
1K	367	0	367	3,794	343	21
3K	1,001	0	1,001	8,866	797	99
10K	2,761	0	2,761	14,578	1278	520
30K	5,470	2	5,448	9,784	532	3,932
100K	8,321	16	8,145	9,147	58	8,509
300K	10,726	33	10,363	10,729	37	10,322

Full—the number of full genotypes that need to cover. Knockout—the number of knockout genotypes that need to cover. Total Cost— 11·Knockout+Full .

**Table 6:** Scenario 3. Comparison of four algorithms: random, greedy, LP, and ILP, to cover 40% from the population of 100K patients by the same 100K donors.

	Cost	Knockout	Runtime (s)
Random	191,000 ± 5,462	15,928 ± 480	500
Greedy	8,339	0	12,600
LP	8,321	13	485
ILP	8,321	16	484

Knockout—the number of knockout genotypes that need to cover. Cost-11 Knockout + number of full genotypes.

#### 3.3.4 Cross-population cellular therapy bio-bank

**Figure 4:**
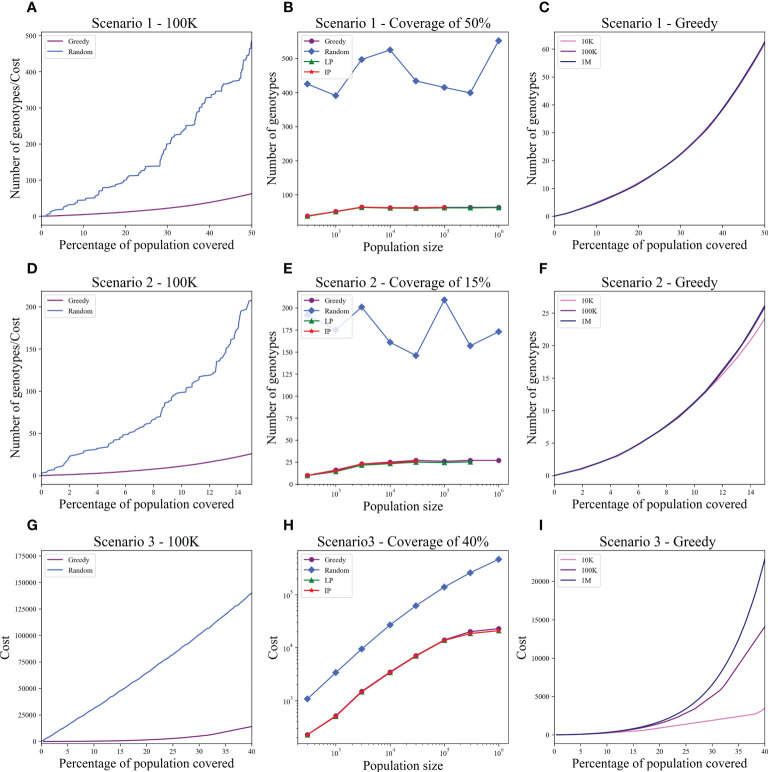
Optimal and random solutions when the donor population is 1 million US donors and the patients are from Israel. All plots are equivalent to **Figure 2**. Each row is a different scenario: upper row - Scenario 1, middle row - Scenario 2, and lower row - Scenario 3. **(A, D, G)** The cost to cover the given percentage from the population of size 100K (x-axis) using two algorithms: greedy and random choice. **(B, E, H)** How many genotypes are needed to cover 50% **(B)**, 15% **(E)**, and 40% **(H)** of differently sized populations represented on the x-axis on a log scale. The y-axis is on a log scale in **(H). (C, F, I)** The number of genotypes needed (y-axis) to cover × percentage of the population (x-axis) in three different population sizes: 10K, 100K, and 1M. While there is a large difference in the number of random donors required (much more here than in **Figure 2**), the number of optimal donors is practically the same. The difference is especially large in Scenario 3. Note that for 1M patients, LP and ILP failed due to memory problem, and for 100K and 300K patients, the ILP did not converge.

While there are differences between populations, the optimal donor group is a small group that may actually be shared between populations. To test for that, we examined the impact of using different populations for the donors and the patients. In all simulations, the donor population is a fixed 1 million donors from the US, and the patient populations are set in different sizes from the Israeli population (as described above). For random donor selection, donors and patients from different populations require many more donors to cover the same patient fraction. In contrast, optimal selection algorithms solve the coverage with a similar number of genotypes as required for donors and patients from the same population in all scenarios tested (**Figure 4**).

## 4 Discussion

The optimal size of donor cell banks is a matter of practical interest. For example, the group at Baylor College of Medicine created a bank of 32 multivirus-specific cell products (transduced with the Ad5f35pp65 vector), of which 18 cell lines were used to treat 50 patients (22). Westmead Hospital created a bank of 31 multiantigen-expanded, multivirus-specific cell products, of which 15 were used to treat 30 patients (21). Memorial Sloan Kettering created a bank of 330 EBV-specific T-cell lines (stimulated with EBV-transformed B-lymphoblastoid cells) and 125 CMVpp65-specific T-cell lines (licensed to Atara Biotherapeutics) (31, 55). Donors for the EBV-specific cell bank were recruited to represent 40 common class 1 HLA alleles that can restrict EBV epitopes. The bank was estimated to cover 95% of the New York population. By contrast, to treat EBV-related post-transplant lymphoproliferative disorders, the Scottish National Blood Transfusion Service performed a simulation using HLA typing from 200 donors from Auckland targeting 304 patients from the East of Scotland renal transplant waiting list, aiming to maximize the number of HLA class 1 and 2 matches and minimize the number of mismatches. Fifteen donors could cover 57% of the patient population and 25 donors could cover 85%, but adding more donors did not significantly increase the coverage. Therefore, the panel size chosen was only 25 (37). In practice, among issued products, there was a median of 3 class 1 matches (range 0–6), 2 class 2 matches (range 0–4), and 5 overall matches (range 2–9) out of 10 loci considered. Clinical responses were positively correlated with number of HLA matches, with 100% of patients with matches at 8 to 10 (of 10) HLA loci responding (38).

These experiences show the wide variety of cell bank building approaches. Our approach facilitates transparency about donor selection and consequently might contribute to reproducibility of outcomes when the “same” products are used in different populations. We recognize that many factors impact the efficacy of off-the-shelf treatment—such as whether the patients are on immunosuppression, the tumor burden, tumor immunogenicity, the presence of particular T or NK cell subsets in the infused product, and the construct of synthetic components. Insofar as HLA match may also impact efficacy, we offer a tool for rationally sizing a bank. These algorithms can readily accommodate additional factors. For example, the bank size can be adjusted to account for the distribution of virus-specific activity in the donor population. For example, seropositivity for CMV (as indicated by CMV IgG) varies widely by age group and geography (56). If the manufacturer is seeking CMV+ donors and knows, for example, that the CMV seroprevalence in the donor pool is 60%, the model could be run with simulations where each potential donor has a 60% chance of being CMV+ and hence being eligible. Similar considerations apply to adjusting the bank size for the rate at which the fully manufactured product fails the release criteria.

An important aspect studied here is the difference between the donor and patient populations. We have shown that when the optimal donors are selected, the number of donors is not significantly affected by differences between the donor and patient populations. This is partly because the algorithms allow rapid identification of the “rarer” donors in the pool who meaningfully increase population coverage, whereas random selection of donors is more likely to select “redundant” donors. Another approach would be to run the algorithm separately for small populations of rare patient genotypes and thus ensure at least a partial coverage.

The algorithms led to a fairly constant number of donors necessary with a population size of about a 100K, even in scenario 3 where it took a somewhat larger population to reach a stabilized number of donors. However, with a random sample, it is much longer until a stable number of donors is reached, if ever.

The current solution is a coverage problem and is not sensitive to the details of the required coverage. We have recently extended the GRIMM, a matching algorithm (46), to allow multiple mismatches. We can use this algorithm to allow for such mismatches. Also, an interesting extension would be to solve the maximum with a constraint that a given sub-population is covered at some fraction.

One aspect of allogeneic cell therapies that we did not address is the possibility of antibody mediated rejection of cells by the patient. The patient may become alloimmunized to foreign HLA through pregnancy or blood transfusions (57). The effect of donor-specific antibodies in HCT (57) and in solid organ transplant (58) is well-studied, but humoral rejection of allo-IECT is not. If it is found to occur frequently or to undermine efficacy; in future work, we could incorporate models of patient alloimmunization that can differ by disease and other demographic factors.

We have simulated a small number of possible scenarios and compared different solutions for the same scenario. In the majority of reported studies, the number of treated individuals is small, and the protocol for choosing donors is not reported. The computational speed and flexibility of the approach presented here will enable better standardization of allo-IECT to elucidate the impact of HLA matching and additional donor-related factors, as both sets of variables can be taken into account in designing the composition of IECT banks. Our approach will enable scaling of current and future studies to the full population using the smallest number of donors, and enable registries like the NMDP to efficiently identify an optimal set of donors for each allo-IECT trial they support.

The code for this analysis is available at https://github.com/sapiris/CAR cells optimization.

## Data availability statement

The original contributions presented in the study are publicly available. This data can be found here: https://github.com/sapiris/CAR_cells_optimization.

## Author contributions

SI performed the analysis and wrote a part of the paper. YL proposed the methodology and wrote a part of the paper. EK and MM developed the clinical scenarios. EK and CS helped with the literature review and with the writing. All authors contributed to the article and approved the submitted version.
